# Specific K39 antibody response and its persistence after treatment in patients with imported leishmaniasis

**DOI:** 10.1007/s00436-015-4801-8

**Published:** 2015-10-28

**Authors:** Ingrid Reiter-Owona, Claudia Rehkaemper-Schaefer, Sandra Arriens, Philip Rosenstock, Kenneth Pfarr, Achim Hoerauf

**Affiliations:** Institut für Medizinische Mikrobiologie, Immunologie und Parasitologie, Universitätsklinikum, Bonn, Germany; Klinik und Poliklinik für Dermatologie und Allergologie, Universitätsklinikum Bonn, Sigmund-Freud Str.25, 53105 Bonn, Germany; Institut Virion/Serion GmbH, 97076 Würzburg, Germany

**Keywords:** Leishmaniasis, K39 ELISA, IFA, Imported leishmaniasis, Diagnosis, Treatment

## Abstract

The sensitivity of a K39 ELISA (Leishmania IgG, Virion/Serion) for the detection of antibodies in patients with imported leishmaniasis was compared with an immunofluorescence assay (IFA), which was applied as “golden standard”. The retrospective study comprised 93 IFA-positive or borderline sera from 42 patients with visceral (*n* = 16) or cutaneous (*n* = 26) leishmaniasis. Patients had acquired infection predominately in the Mediterranean area or the Middle East. The *Leishmania* species (*Leishmania donovani*/*infantum*, *Leishmania tropica*, *Leishmania major*) were identified by real-time PCR. The majority (94 %) of first samples from patients with visceral leishmaniasis (VL) tested positive by K39 ELISA. Antibody levels ranged from low to very high (33.19–1990.00 U/ml; median 596.66 U/ml) but did not correlate with the respective IFA titers. High K39 ELISA values correlated with acute infection in immunocompetent individuals. K39 antibodies declined in all individuals after clinically successful therapy, but time to seronegativity varied considerably (51 weeks to >6 years). In patients with cutaneous leishmaniasis (CL), the sensitivity of the K39 ELISA was low (23 %) compared to IFA (92 % positive). Antibody levels ranged from low to medium (10.85–524.77 U/ml; median 19.77 U/ml). The highest antibody concentrations were seen in *L. infantum*-infected individuals. Summarizing, a high K39 ELISA value indicates active VL. The assay is, like IFA, not a measure for effective therapy but may support post-treatment monitoring. Low level positivity can indicate subclinical, previous or clinically cured VL or even CL. The K39 ELISA can supplement highly sensitive screening tests in the diagnosis and follow-up of imported leishmaniasis.

## Introduction

Leishmaniasis is an infectious disease, caused by protozoic parasites of the genus *Leishmania*. The disease occurs predominantly in tropical and subtropical climate zones.

Depending on the infecting species and the hosts’ immune reaction, *Leishmania* can induce different clinical manifestations, which are referred to as cutaneous leishmaniasis (CL), mucocutaneous leishmaniasis (MCL) or visceral leishmaniasis (VL). Cutaneous leishmaniasis of the Old World is frequently caused by *Leishmania tropica*, *Leishmania major* or *Leishmania aethiopica* and is known as a self-limiting disease. Infections with *Leishmania donovani*, *Leishmania chagasi* and—in Europe—*Leishmania infantum* may also induce CL but tend to induce visceral disease (VL; also known as kala-azar).

It is estimated that the annual worldwide incidence is approximately 0.2 to 0.4 million cases of visceral and 0.7 to 1.2 million cases of cutaneous leishmaniasis (Alvar et al. 2012). In north European countries, where leishmaniasis is regarded as an emerging disease, it is still rare and predominately imported (Harms et al. 2003; Malik et al. 2006). In Germany, 16 cases of VL and 23 cases of CL were recorded in the period between 2001 and 2004, the majority of which originate from Mediterranean holiday destinations (Weitzel et al. 2005).

Due to the non-specific symptoms of CL and VL, diagnosis is often delayed in non-endemic countries (Gradoni 2013). Diagnosis is based on direct pathogen detection methods, such as microscopy, in vitro culturing or molecular biological methods like PCR. Also, the detection of specific antibodies by various serological methods is common for MCL and VL, but is less recommended for CL because of rather low sensitivity.

For cutaneous leishmaniasis, the diagnostic value of serology depends on the causative *Leishmania* species, which differ in eliciting immune response (Romero et al. 2005) but also on the immune response of the host and the sensitivity of the assay used. Usually, antibody detection is most reliable in immunocompetent individuals with VL and is used as a fast, low invasive and efficient diagnostic method. In recent years, assays based on a defined antigen, the kinesin-like protein K39 derived from *L. chagasi*, are increasingly applied in endemic countries for the detection of patients with visceral disease. The assays are described as highly specific and sensitive (Maalej et al. 2003; Chappuis et al. 2007; Maia et al. 2012) and should indicate successful therapy.

Due to the limited number of imported cases, K39-based assays are rarely applied in non-endemic countries. In this study a K39-antigen based ELISA (SERION ELISA classic *Leishmania* IgG) was tested for its suitability to detect antibodies in cases with leishmaniasis imported into Germany for the first time. The retrospective study, which included 42 patients with confirmed visceral or cutaneous infection, and antibody response confirmed by IFA, should reveal to which extent the serological results provide information on the kind of clinical manifestation, the severity of the disease, and successful treatment.

## Material and methods

For the present study, 93 serum samples from 42 patients with clinical and laboratory diagnosis of visceral leishmaniasis (*n* = 16) or cutaneous leishmaniasis (*n* = 26) were selected from archived material. The selection criteria were clinical symptoms for—or history of exposure to *Leishmania spp*., identification of the parasite in a clinical sample or follow-up after specific therapy and seropositivity by IFA. The serum samples, collected at different clinical sites in the context of the routine examination of returnees or immigrants from endemic areas, were sent to the Institute for Microbiology, Immunology and Parasitology for *Leishmania* serology. The collection period was from 2006–2014.

The mean age of patients with VL was 47 years (range 1.5–80 years), the mean age of patients with CL was 44 years (range 2–84 years). The male:female ratio was 12:4 (VL) and 17:9 (CL), respectively.

For 37 patients, a first sample was available before initiation of treatment (11 patients with VL, 26 patients with CL). From four VL patients, the first sample was taken after the onset of treatment. After treatment follow-up samples were available from nine VL patients (min. 13 weeks, max. 145 weeks) and six CL patients (min. 13 weeks, max. 106 weeks).

Serology: All serum samples had been tested individually at the time of collection by an indirect fluorescence assay (IFA) and were subsequently stored at−25 °C. Samples were thawed to perform the K39 ELISA and for repeated IFA testing in case of discrepant or unclear results to ensure continued seroreactivity and stability.

IFA: The in-house assay was carried out using amastigotes from a Mediterranean *L. infantum* isolate (strain B). Cryosections with amastigotes were prepared with liver or spleen taken from previously infected golden hamsters. The IFA was performed using standard procedures. Serum dilution (twofold) started at 1:10. For staining, a FITC-labelled anti-human Ig-conjugate (BioMérieux, France) was used at a dilution of 1:100. Titers of 1:10 were considered as borderline, >1:10 as positive for people without travel history to a country endemic for Chagas. The IFA, which is highly sensitive for the detection of Old World Leishmaniasis, served as “golden standard”. High titers are commonly associated with visceral infection, moderate or low titers with asymptomatic or cutaneous infection.

K39 ELISA: For ELISA analysis, the commercially available SERION ELISA classic *Leishmania* IgG (Virion\Serion GmbH, Würzburg, Germany) was used in accordance to the manufacturer’s instructions. The assay is based on a preparation of K39 and allows for quantitative data interpretation. Antibody activities of 10–15 U/ml are evaluated as borderline, values > 15 U/ml are considered positive. Samples with a borderline or positive result were confirmed by repeated testing.

### Real-time PCR

Subgenus or species-specific primers and four TaqMan hybridization probes were designed to detect and differentiate *L. major*, *L. donovani*/*infantum*, *L. braziliensis* and *Leishmania tropica* from one another in patient material based on the glucose-6-phosphate sequences of these species (Table 1). Two forward primers and a single reverse primer amplify a region with differences that can be detected with four TaqMan probes. In a total volume of 20 μl, 5 μl of DNA was amplified using the Quantitect Multiplex NoRox Kit (Qiagen, Hilden, Germany) in accordance to the manufacturer’s protocol with 200 nM of each forward and reverse primer and 50 nM of each hybridization probe. Samples were amplified and fluorescence acquired using a Rotorgene Q 5-plex real-time machine (Qiagen). The following protocol was used: 95 °C 15 min, 55 cycles of 94 °C 10 s, and 62 °C 30 s. Fluorescence was acquired at the end of the binding/extension step. Plasmids (1 × 10^5^ copies/μl) containing each species-specific sequence were used as positive controls for the PCR. To control for DNA extraction, *L. donovani* cultivated at 25 °C in supplemented L-15 Leibovitz Medium (Biochrom, Berlin, Germany) was extracted in parallel with the patient samples using the QIAamp DNA Mini Kit in a QIACube. The identities of all strains had been verified by zymodeme analysis and RFLP-PCR (Schoenian et al. 2001).

**Table 1:** Primer and hybridization probe sequences for *Leishmania spp.* glucose-6-phosphate (GenBank: XM_003859118.1, XM_003392245.1, AY974218.1, XM_001681607.1, and EU053104.1) and *Mus musculus* interferon-gamma (mIFN-γ; GenBank: EF423643.1)

Name	Sequence 5′-3′	5′ modification	3′ modification
braz/inf F1	ccgaccaaagccagcatcat		
braz/inf R1	tactctgtgtggtccgccat		
maj/tropF1	acgccagcatcatcggcaag		
trop TQP	tttgtggagttcttgactggc	HEX	BHQ-1
do/inf TQP	tttgtggagttcctgactggc	Cy5	BBQ-650
maj TQP	tttgtggaattcctgacaggcg	FAM	BHQ-1
braz TQP	aattctgctccaccggcgctga	ROX	BBQ-650
mIFNg-FW	tcaagtggcatagatctggaagaa		
mIFNg-RV	tggctctgcaggattttcatg		
mIFNg-TQP	tcaccatccttttgccagttcctccag	DY-681	BBQ-650

Serum and plasma were eluted in 50–75 μl of elution buffer while whole blood was eluted in 100 μl of elution buffer. Extraction of biopsies was performed using the QIAamp DNA Mini Kit including a prior step of digestion with proteinase K. Samples were used immediately for real-time PCR or frozen until use at−20 °C.

DNA: As a control for inhibitors in the DNA extracted from patient blood, a second reaction combining sample DNA with a plasmid containing a fragment of *Mus musculus* interferon-gamma (mIFN-γ; GenBank: EF423643.1) was performed in parallel (Albers et al. 2012). In short, in a total volume of 20 μl, 5 μl of sample DNA and 2 μl of mIFN-γ plasmid (1 × 10^6^ copies/μl) were amplified using the Quantitect Multiplex NoRox Kit (Qiagen) in accordance to the manufacturer’s protocol with 200 nM of forward and reverse primers and 50 nM of the hybridization probe (Table 1). Cycling conditions were the same as for the *Leishmania spp.* PCR. Samples were considered inhibited if C_t_ of the mIFN-γ plasmid was detected ≥3 cycles later than the C_t_ for the water control. By this method, the DNA from this patient cohort did not contain inhibitors that needed to be controlled for by dilution or other means.

## Results

IFA titers of all samples retested during the study were in concordance with values achieved at the time of primary testing.

Visceral leishmaniasis: All but one of the first samples from 15 patients with VL tested positive by K39 ELISA. The majority of the patients was immunocompetent and got infected in the Mediterranean area with *L. infantum* (Table 2). For a few patients, travel history or the clinical symptoms were not reported or the *Leishmania* species remained unknown (samples for PCR not available).

**Table 2:** Characteristics of 15 patients with visceral leishmaniasis

Patients	K39 ELISA result qualitative	
	Positive	Negative
Travel history or origin	9	1
Mediterranean area	1	–
Uzbekistan	1	–
West Africa Unknown	3	–
Immune status Competent Suppressed	11 1 2	– 1 –
HIV-coinfection		
Clinical symptoms	9	1
VL	3	–
CL + VL (disseminated) Unknown	2	–
*Leishmania* species	9	1
*L. infantum*	1	–
*L. major* Unknown^a^	4	–

^a^No adequate clinical sample available for PCR

**Fig. 1:**
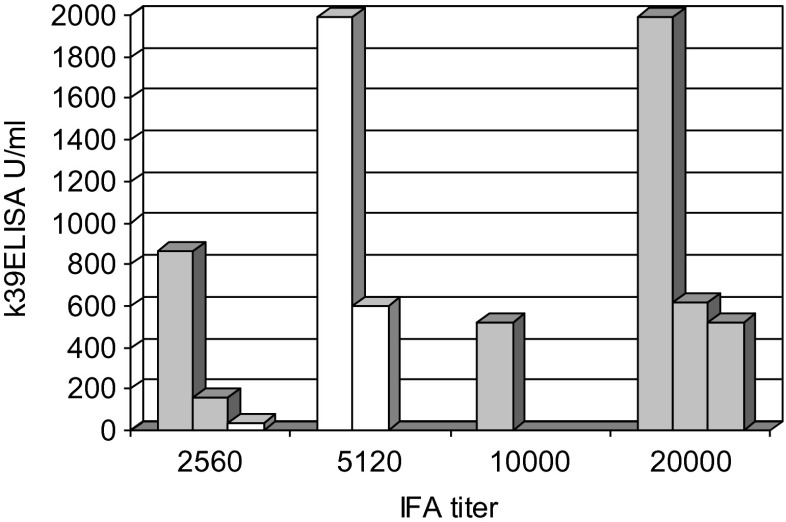
K39 ELISA and IFA antibody concentration in nine immunocompetent patients with visceral leishmaniasis before treatment. *Grey columns*: infection with *L. infantum*; *white columns*: *Leishmania* species not determined

Regarding the immunocompetent VL patients with a first sample before treatment (*n* = 9) all IFA titers were high (2560–20,000, median 10,000), whereas K39 ELISA results (IU/ml) ranged from low to very high (33.19–1990.00; median 596.66) without a clear correlation to IFA values. In six patients, an *L. infantum*-infection was determined by PCR, in three patients there was no appropriate sample available for PCR (Fig. 1).

The four immunosuppressed patients with clinical signs of disseminated and visceral leishmaniasis showed varying IFA and K39 ELISA values (Table 3). From all patients, at least one clinical sample with a positive PCR result was available in which *Leishmania*-DNA was detected and specified. Three patients were infected with *L. infantum*. One individual with a positive bone marrow and blood sample (*L. infantum*) after a history of extended steroid application (Tables 3 and 4) remained negative by K39 ELISA. Of special interest is the HIV co-infected individual with disseminated *L. major*-infection, in whom the highest IFA titers in combination with low ELISA values were seen. The highest ELISA values occurred in a patient infected with *L. infantum* who showed severe skin manifestations and clinical signs for VL but very low parasite concentration in the bone marrow (PCR weakly positive, microscopy negative).

**Table 3:** *Leishmania* antibodies and real-time PCR results in immunosuppressed patients with clinical signs of visceral (VL) or disseminated leishmaniasis (DL)

Patient No. (manifestation)	Underlying disease	Result real-time PCR		Result serology	
		Sample	Species	IFA titer	K39 ELISA U/ml
1 (DL)	HIV+	Blood+, skin+	*L. major* *L. major*	20,000 (+)	16.02 (+)
2 (VL)	HIV+	Blood+	*L. infantum*	320 (+)	56.77 (+)
3 (DL)	Kidney transplantation	Skin+, bone marrow (+/−)	*L. infantum* *L. infantum*	640 (+)	1977.72 (+)
4 (VL)	Lupus erythematosus	Blood+, bone marrow+	*L. infantum* *L. infantum*	160 (+)	1.82 (−)

**Table 4:** Antibody response in five individuals with visceral leishmaniasis after initiation of treatment

Patient *Leishmania* species	Weeks posttreatment	Result K39 ELISA		Result IFA	
		U/ml	Qual.	Titer	Qual.
B *Leishmania infantum*	0	616.58	+	20,000	+
	16	66.5	+	5120	+
	43	25.69	+	2560	+
	66	56.67	+	1280	+
	95	15.77	+	640	+
	122	7.64	Neg.	320	+
	145	7.26	Neg.	320	+
F *Leishmania infantum*	0	516.3	+	20,000	+
	6	683.8	+	5120	+
	12	635.82	+	5120	+
	23	419.03	+	2560	+
	46	248.02	+	1280	+
	75	169.33	+	640	+
M *Leishmania major*	0	16.02	+	20,000	+
	12	46.56	+	20,000	+
	39	47.39	+	2560	+
	46	20.41	+	1280	+
	84	19.32	+	640	+
	90	13.93	Neg.	640	+
J *Leishmania infantum*	0	159.41	+	2560	+
	5	95.54	+	640	+
	13	21.12	+	320	+
L *Leishmania infantum*	0^a^	56.77	+	640	+
	23	23.64	+	640	+
	39	30.99	+	640	+
	51	9.99	Neg.	320	+

^a^Follow-up after the third treatment schedule

Follow-up after treatment: Five patients were regularly followed up after different treatment regiments from 13 (minimum) up to 145 (maximum) weeks. After clinically successful therapy, a decline of K39 antibodies was detectable in all individuals but it followed an individual pattern (Table 4). The first negative result was recorded for patient L (HIV+) at 51 weeks after the third treatment schedule with glucantime. Patient M (HIV+) was ELISA negative at 90 weeks after treatment with pentamidine, whereas patient B was negative only 122 weeks after treatment with liposomal amphotericin B. The remaining individuals, children at the age of 17 months (J) and 15 years (F), respectively, had not returned to ELISA negativity at their final test at 13 or 75 weeks after treatment with liposomal amphotericin B. Nevertheless, significant drops in the K39 antibody levels were observed in both patients.

From two male patients with a history of VL, first serum samples were tested 8 and 2 years, respectively, after the onset of treatment. At that time they were clinically asymptomatic and showed similar IFA values (1:640, 1:320) but a negative or a moderate K39 ELISA result. At the end of a subsequent screening at regular intervals for another 4 years, the first individual (HIV+, PCR−) remained ELISA negative (IFA 1:320), the second individual ELISA moderately positive (IFA 1:80).

Cutaneous leishmaniasis: When studying the 39 sera from 26 patients with CL (one patient was asymptomatic after spontaneous healing of a very small skin lesion) the majority of sera tested negative by ELISA. IFA values ranged from 1:10 (borderline) to 1:320. Infection was acquired mainly in the Mediterranean area (*L. infantum*) and Syria (*L. tropica*). A low number of lesions (1–3) existed in 86 % of the patients for whom clinical reports were available (Table 5). As the exact size of the lesions was not recorded for most of these patients, this specific parameter was not included.

**Table 5:** Characteristics of 25 patients with clinical signs of cutaneous leishmaniasis (CL) and a positive IFA-result (golden standard)

Patients	K39 ELISA	K39 ELISA
	positive/borderline	negative
Travel history or origin		
Mediterranean area	2	6
Syria	3	7
Iran	1	1
Tunisia	–	1
Bolivia	–	1
Turkey + Sinai	1	–
Unknown	–	2
Immune status		
Competent	7	18
Suppressed	–	–
Clinical symptoms		
CL	7	17
Unknown	–	1
CL: number of lesions		
1	4	8
2–3	2	5
>3	1	2
Unknown	–	3
*Leishmania* species		
*L. infantum*	2	6
*L. tropica*	4	6
Unknown	1	6

Only six patients (23 %) were found ELISA positive or borderline in their first sample (Table 6). Antibody levels ranged from low to medium (10.85–524.77 U/ml; median 19.77 U/ml). Four individuals were infected with *L. tropica*, two individuals with *L. infantum*. None of the patients had clinical symptoms for visceral infection. The highest antibody concentrations were seen in *L. infantum*-infected patients with single or multiple lesions, respectively.

**Table 6:** Borderline or positive K39 ELISA in patients with cutaneous leishmaniasis

Patient No.	Clinical manifestation	IFA (titer)	K39ELISA (U/ml)	Real-time PCR skin
1	1 lesion lower arm	Positive	Borderline	Positive
		(80)	(10.58)	(*L. tropica*)
2	1 lesion lower lip	Positive	Borderline	Positive
		(40)	(12.09)	(*L. tropica*)
3	1 lesion upper lip	Positive	Positive	Positive
		(40)	(24.08)	(*L. tropica*)
4	2 lesions lower arm	Positive	Positive	Positive
		(40)	(15.27)	(*L. tropica*)
5	1 large lesion, elbow	Positive	Positive	Positive
		(160)	(51.46)	(*L. infantum*)
6	10 small lesions, face	Positive	Positive	Positive
		(5120)	(524.77)	(*L. infantum*)

**Fig. 2:**
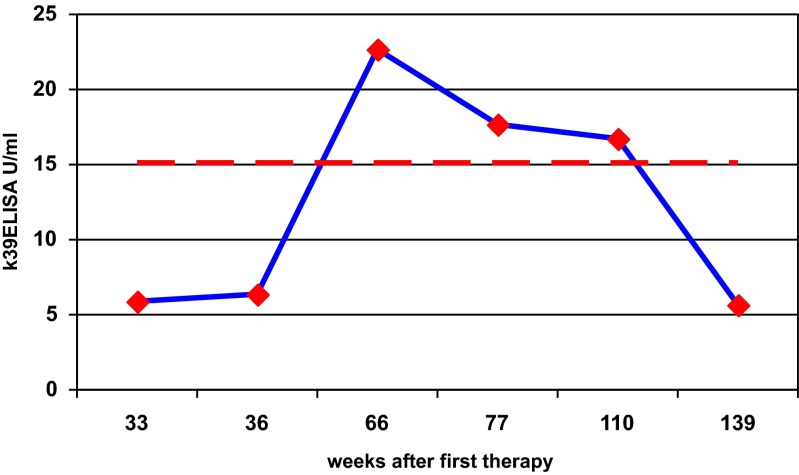
Seroconversion after first treatment in a patient with cutaneous leishmaniasis and three facial lesions before treatment. *Broken line* = cutoff

Follow-up after treatment: Follow-up samples were available from five (initially ELISA-negative) patients (period of follow-up minimum 13 weeks, maximum 139 weeks). In one patient, a rise of anti-K39 antibodies was documented 12 weeks after the third treatment schedule ((1) freezing, (2) miltefosin, (3) ketoconazole) when the most persistent of the initially three facial lesions started to regress in size. Low level antibodies remained detectable for a period of 44 weeks until the lesion was nearly healed (Fig. 2). The increase in antibodies detected by ELISA was not accompanied by a rise of IFA antibodies (1:80).

## Discussion

Various serological tests have been developed for the detection of specific antibodies against *Leishmania*. Generally in VL patients, serological assays are known to be sensitive for diagnosis (Chappuis et al. 2007), whereas in cases of CL serology is considered to be of low predictive value due to low antibody concentration. There is no consensus regarding the diagnostic use of the available tests in a specific clinical situation except for the rK39 antigen-based assays. rK39 positivity is usually related to the presence of a clinically symptomatic visceral infection (Srividya et al. 2012). The antigen is part of a large kinesin-related protein expressed predominantly by amastigotes. The antigen is shared by members of the *L. donovani* complex (*L. chagasi*, *L. donovani*, *L. infantum*) (Burns et al. 1993). Burns et al. (1993) reported a high prevalence of antibodies to K39 in VL patients from different geographically regions, whereas patients with CL or mucocutaneous leishmaniasis remained negative. Since its first description, the antigen has been widely applied either in a strip test or in an ELISA format and is sufficiently validated for field use in areas endemic for VL (Chappuis et al. 2007; Maia et al. 2012) or for immigrants from endemic areas with VL (El-Moamly et al. 2012) but there is little experience on its performance in people from non-endemic areas who got infected abroad.

In our study we examined the characteristics of a commercially available K39 ELISA (antigen from *L. infantum*) in patients with imported VL and CL in comparison to the IFA, which is known to be of high diagnostic accuracy (Chappuis et al. 2007). An amastigote-based IFA, similar to the one used in this study, was found to be at least as sensitive as the direct agglutination test (El Harith et al. 1989).

We could confirm that all immunocompetent patients with clinical signs of visceral disease and a high IFA antibody concentration were also positive by ELISA. A good agreement between DAT and rK39 ELISA is known from symptomatic patients with VL but it is poor in subclinically infected individuals (Srivastava et al. 2013). In our study, there was no correlation between the quantitative values of both tests which use antigens derived from the same developmental stage of the parasite (amastigotes). The quantitative ELISA results, which varied from very low (33.9 U/ml) to very high (>1990 U/ml) suggest that the K39 expression or recognition is different in individual VL patients of German origin who were infected with *L. infantum* in the Mediterranean area. A similar observation is reported for French VL patients also infected with *L. infantum* from the Mediterranean area (Abass et al. 2015).

A variable performance of the rK39 dipstick tests in VL patients from different geographical areas is well known (Maia et al. 2012; TDR 2010). In contrast, a qualitative rK39 ELISA applied in a recent study (Abass et al. 2015) was most sensitive in immunocompetent patients from all endemic regions (96–100 %). The highest antibody concentrations, however, were found in patients from India, compared to patients from Sudan and France.

A reduced sensitivity (81.8 %) of a rK39 ELISA is reported for French HIV co-infected patients (Abass et al. 2015), whereas in Italian HIV+ persons the ELISA was found more sensitive than an IFA based on promastigotes (Houghton et al. 1998). Data from our small cohort of immunosuppressed individuals indicate that the K39 ELISA values may remain very low or even under the cutoff in heavily infected individuals although IFA antibodies were at high to medium level.

We could not confirm that the K39 ELISA applied in this study was generally better in detecting high than low level IFA antibodies in VL patients as mentioned elsewhere (Abass et al. 2015). Whether differences in antibody production to the kinesin-related antigen are due to the stage of infection or parasite load could not be evaluated in our study.

Today, the follow-up of rK39 antibodies after treatment is no longer considered as an adequate control measure for effective therapy (Vallur et al. 2015). However, a serological follow-up of our patients with VL after specific treatment revealed a decline of K39 and IFA antibodies in all successfully treated individuals within the first 12 months as reported elsewhere (Gidwani et al. 2011), but the antibody decrease followed an individual pattern. The earliest documented seronegativity in an immunocompetent individual was at 122 weeks (>2 years) after onset of treatment. One patient even remained ELISA positive for more than 6 years without clinical signs for VL. In contrast to studies performed in endemic areas, where a large proportion of post kala-azar patients remains seropositive (rK39 ELISA) after cure (Srivastava et al. 2013), some for more than 15 years (Gidwani et al. 2011), in our study group a repeated exposure to *Leishmania* was not probable. It is remarkable that in one HIV+ individual antibody levels had dropped under the detectable level after treatment at times when *Leishmania* DNA from peripheral blood was still detectable by PCR. This finding argues against the K39 ELISA as an instrument for monitoring immunosuppressed individuals after treatment as suggested elsewhere (Houghton et al. 1998). In contrast to other authors (Cota et al. 2012; Houghton et al. 1998) we found the IFA format applied here was an appropriate test to confirm leishmaniasis in immunocompetent and immunosuppressed individuals who were infected in the Old World. However, study results from different groups may be influenced not only by the strain and the stage of the parasite used for antigen preparation but also by the characteristics of the tested populations (Cota et al. 2012).

As expected, the K39 ELISA was less sensitive in patients with CL than in patients with VL. Only 23 % of the patients with CL were positive or borderline by ELISA, which is in concordance with a recent study were 29 % of US servicemen (*n* = 59) with active CL had developed antibodies against rK39 detectable in an ELISA format. The majority of them were infected with *L. major* (20) and only two with *L. tropica* (Hartzell et al. 2008), whereas patients from our study were infected with *L. tropica* and *L. infantum*, respectively. For the US servicemen, it was concluded that seropositivity occurs when more and larger lesions exist, whereas in our study there was a tendency that infection with *L. infantum* induces a more pronounced antibody response than infection with *L. tropica*. In both studies, antibody concentration was moderate and only few individuals showed elevated ELISA values at levels comparative to patients with VL. The K39 ELISA used in our study was better in detecting high level IFA antibodies in CL patients but those who were negative by K39 ELISA did not generally have low IFA titers. The IFA format used in this study proved to be very sensitive for the diagnosis of CL of the Old World and antibodies persisted up to 24 months after *L. tropica*-infection, which is in concordance with persistence up to 27 months described earlier (Menzel and Bienzle 1978). We could exclude that ELISA positive CL patients in our study had clinical signs for visceral infection. Also, seroconversion which occurred in one patient only at the end of three consecutive treatment trials was associated with the resolution of clinical symptoms and not with a progression of the disease.

With a dipstick assay, which in CL is about two third less sensitive than the ELISA format (Hartzell et al. 2008), anti-rK39 antibodies were not detected in Turkish children with small, non-ulcerating lesions due to *L. infantum* (Svobodová et al. 2009), whereas 31 % of Indian patients with CL due to *L. donovani* reacted positive (Sharma et al. 2009), a difference which may be explained by the generally higher antibody level in the Indian population.

Today, it is well known that a positive anti-K39 response is not necessarily associated with active visceral infection. Antibody prevalence in areas endemic for VL may be up to 34.4 % in the asymptomatic population (Hantosh and Al Lami 2013).

In conclusion, we could confirm that a high level of K39 antibodies can indicate an active visceral or disseminated infection. In immunocompetent individuals, the K39 assay allows for reliable detection of visceral leishmaniasis and can support the post-treatment monitoring. However, low level positivity, may be the result of a subclinical, a previous or a clinically cured VL or even indicate cutaneous infection and can persist up to 6 years after adequate VL treatment. Srivastava et al. (2013) noted that the use of a second test (DAT) could improve the poor detection rate of the rK39 ELISA for asymptomatic individuals in areas endemic for VL. In non-endemic countries the K39 ELISA tested here can give additional information when it is run in parallel with a highly sensitive screening test like the IFA.
